# Fra2 Overexpression in Mice Leads to Non-allergic Asthma Development in an IL-13 Dependent Manner

**DOI:** 10.3389/fimmu.2018.02018

**Published:** 2018-09-05

**Authors:** Anna Gungl, Valentina Biasin, Jochen Wilhelm, Andrea Olschewski, Grazyna Kwapiszewska, Leigh M. Marsh

**Affiliations:** ^1^Otto Loewi Research Center, Medical University of Graz, Graz, Austria; ^2^Ludwig Boltzmann Institute for Lung Vascular Research, Graz, Austria; ^3^Department of Internal Medicine, Universities of Giessen and Marburg Lung Center, Giessen, Germany; ^4^German Center for Lung Research, Justus-Liebig University, Giessen, Germany

**Keywords:** extrinsic asthma, AP-1, airway hyperresponsiveness, Fra-2, mucus hypersecretion

## Abstract

**Background:** Asthma is a complex chronic inflammatory disease characterised by airway inflammation, remodelling and hyperresponsiveness (AHR). Members of the AP-1 transcription factor family play important roles in the activation of the immune system and the control of cellular responses; however, their role in the development of asthma has not been well studied. We aimed to investigate the role of the lesser known AP-1 family member, Fra2 in experimental asthma.

**Methods:** Phenotypic characterisation and gene expression profiling was performed on Fra2 (TG) overexpressing and wild-type mice. The efficacy of therapeutic interventions in regulating the Fra2 phenotype was determined.

**Results:** Transcriptional profiling of TG mice revealed a high abundance of regulated genes associated with airway remodelling, inflammation and mucus production. A concomitant increase in peribronchial collagen deposition, smooth muscle thickening and mucus production was observed. TG mice possessed increased inflammatory infiltration in the lung, predominantly consisting of eosinophils and T-cells and elevated expression of Th2 cytokines and eotaxin. Furthermore, TG mice possessed severe AHR in response to increasing doses of methacholine. Glucocorticoid treatment led to a partial improvement of the asthma phenotype, whereas blockade of IL-13 via neutralising antibodies ameliorated AHR and mucus production, but had no effect on collagen deposition.

**Conclusion:** We here describe a novel model for non-allergic asthma that does not require the application of exogenous allergens, which mimics several key features of the disease, such as airway inflammation, remodelling and hyperresponsiveness. Fra2 may represent a key molecule coordinating multiple aspects of asthma pathogenesis.

## Introduction

Asthma is a chronic lung disease that affects over 300 million people worldwide (1). Here airway remodelling manifests as mucus cell metaplasia, smooth muscle thickening and sub-epithelial fibrosis, which gives rise to airway obstruction and hyperresponsiveness (AHR) (2). The most common form of asthmatic airway disease is atopic (extrinsic) asthma, which is driven by a Th2 allergic response (3). Elevated levels of eosinophils, T-cells and the Th2 cytokines, interleukin (IL)-4, IL-5 and IL-13, cause airway remodelling and bronchoconstriction (4). Although, IL-4 and IL-13 share a functional receptor complex (IL-4Rα/IL-13Rα1), they possess distinct roles in mediating allergic asthma *in vivo*. IL-4 is important in the initiation of allergic responses (Th2 cell proliferation or IgE synthesis), while IL-13 is more important for the effector phase (induction of AHR, mucus production and airway smooth muscle hyperplasia) (5). IL-13 promotes mucus hypersecretion by inducing expression of several transcription factors such as SPDEF (SAM pointed domain containing ETS transcription factor), which are important for the differentiation of Club/Clara cells to goblet cells, and additionally regulates genes important for mucus production and secretion (e.g., *Muc5ac and Clca1)* (6–8).

In addition to eosinophilic/Th2-driven allergic asthma, several other subtypes of asthma have been described, including neutrophil-predominant or mixed eosinophilic/neutrophilic subtypes, which often do not respond to classical glucocorticoid treatment (9). The underlying basis of steroid resistance in certain asthma subtypes is still unclear. Some hypothesized mechanisms include increased presence of certain cytokines (such as IL-13 or IL-17), decreased levels or altered DNA-binding affinities of the glucocorticoid receptor (GR), or increased activation of pro-inflammatory transcription factors, such as NF-kB or AP-1 (10, 11).

Investigation into the underlying mechanisms of asthma pathogenesis often relies on animal models, which also form the foundation of early pre-clinical testing. As mice do not spontaneously develop asthma, an asthmatic phenotype must first be induced (12). Several different protocols exist that induce acute allergic airway inflammation in mice. These traditionally use intraperitoneal sensitisation of an allergen (typically ovalbumin) and the non-physiological adjuvant alum to promote the development of a Th2-driven immunological response (12, 13). Several adjuvant free mouse models exist including sub-cutaneous ovalbumin sensitisation (14) or direct exposure of the lung to house dust mite, aspergillus or cockroach aeroallergens (15–17). All these models rely on the application of an exogenous allergen to induce acute allergic airway inflammation and imitate early-onset atopic asthma (12). Currently, there is a lack of other pre-clinical models, which represent different asthma phenotypes, such as non-atopic asthma.

The activator protein-1 (AP-1) transcription factor complex has important roles in cell proliferation, differentiation and regulation of inflammatory processes (18). AP-1 is present as both a homo- or heterodimeric complex, which consists of different combinations of the Jun (c-Jun, JunB, JunD) and Fos (c-fos, FosB, Fra1, Fra2) subunits. AP-1 can be activated by multiple stimuli, including cytokines such as IL-6 (19) or IL-13 (20); intrinsic danger signals (21) or growth factors e.g. endothelin-1 or connective tissue growth factor (22, 23). In turn the typical Th2 cytokines, IL-4, IL-5, and IL-13 possess AP-1 binding sites in their promoter regions and their expression is influenced by AP-1 activity (24–27). In asthma, increased AP-1 DNA binding has been observed in bronchial fibroblasts isolated from asthmatic patients compared to non-asthmatic controls (28). Furthermore, inhibition of AP-1 via decoy oligonucleotides has been shown to attenuate OVA-induced experimental asthma (29). However, the contribution of individual AP-1 subunits in the development of asthma is still unclear.

In mice ectopic overexpression of the AP-1 subunit Fra2 leads to an age-dependent increase in perivascular and peribronchial inflammation in the lung. In older mice, overexpression ultimately leads to the development of fibrosis, predominantly affecting the lung, but also can affect other organs including the skin (30, 31). Here we have used an unbiased gene expression profiling approach to analyse the underling pathomechanisms that could mediate the TG phenotype. Importantly, this analysis was performed at an early time-point when mice do not exhibit any overt phenotype. Gene profiling revealed a high proportion of regulated genes that were associated with asthma susceptibility and airway remodelling. We therefore hypothesised that Fra2 TG mice may develop experimental asthma. To this end we thoroughly investigated chronic airway disease in these mice and examined the importance of IL-13 and steroid treatment in regulating this phenotype.

## Materials and methods

### Animals

Fra2 transgenic (TG) mice were obtained from Erwin Wagner, Research Institute of Molecular Pathology, Vienna (30) and bred in house. Mice were maintained under specific pathogen free conditions in isolated ventilated cages with 12 h light/dark cycles. Water and chow were supplied *ad libitum*. All mouse experiments met EU guidelines 2010/63/EU and were approved by the Austrian Federal Ministry of Science, Research and Economics. All measures were taken to keep animal suffering to a minimum. The characterisation of the TG phenotype was performed in two independent experiments with five to seven mice per group. To block IL-13 activity, 150 μg of rat anti-mouse IL-13 (IgG1κ) antibody (clone eBio1316H, ThermoFisher Scientific, Waltham, MA) per mouse were injected intraperitoneally twice a week (32). Control mice received 150 μg rat IgG1κ isotype control (clone RTK2071). To investigate the effect of glucocorticosteroid treatment, 10 μg budesonide (Pulmicort® suspension, AstraZeneca, Cambridge, UK) per mouse were applied intranasally three times a week. Both treatments were started in 10–11 week old mice. Analysis of AHR and organ collection was performed after 6 weeks of treatment. Anti-IL-13 and glucocorticoid treatments were performed once with five to eight animals per group.

### Microarrays

Total RNA was isolated from the lungs of 16-week-old Fra2 TG and WT littermate control mice (eight animals per group) using the RNeasy Mini kit (Peqlab, Erlangen, Germany). 200 ng of total RNA was preamplifed and labelled with Cy5 using the Low-input QuickAmp Kit (Agilent Technology, Santa Clara, CA) according to the manufacturer's instructions. Hybridizations were performed for 18 h at 42°C on Agilent 6x80K mouse microarrays in Agilent hybridization chambers. The data were analysed using the limma package in R. Intensity values were background-corrected and quantile normalized. Significance of differential expression was estimated using moderated t-statistics as previously described in full (33). Regulated genes, defined as genes with a fold change >2 and a significance of *p* < 0.05, were screened for association with asthma using UniProt Knowledgebase and by conducting a PubMed search using the search terms “asthma” and “airway disease” together with the gene names or corresponding aliases.

### *In silico* transcription factor binding site analysis

Transcription factor binding site analysis was performed with the ConTra v3 web server [as described in (34)]. Promoter regions, up to 1 Kb 5′ of the transcription start site of genes related to mucus production and secretion (Table 1) were analysed with *mus musculus* as reference organism. The positional weight matrix motif V$AP1_CM00199 (TRANSFAC20113 database) was selected for visualization with a core stringency of 0.90 and similarity stringency of 0.75.

**Table 1:** Genes used for *in silico* transcription factor binding site analysis.

**Gene symbol**	**Gene name**	**Reference number**	**Ensembl gene ID**
*Agr2*	chloride channel calcium activated 1	NM_011783	ENSMUSG00000028255
*Clca1*	anterior gradient 2	NM_009899	ENSMUSG00000020581
*Ern2*	endoplasmic reticulum (ER) to nucleus signalling 2	NM_012016	ENSMUSG00000030866
*Fcgbp*	Fc fragment of IgG binding protein	NM_001122603	ENSMUSG00000047730
*Galnt12*	polypeptide N-acetylgalactosaminyltransferase 12	NM_172693	ENSMUSG00000039774
*Itln1*	intelectin-1a-like	NM_010584	ENSMUSG00000038209
*Muc5ac*	mucin 5, subtypes A and C, tracheobronchial/gastric	NM_010844	ENSMUSG00000037974
*Muc5b*	mucin 5, subtype B, tracheobronchial	NM_028801	ENSMUSG00000066108
*Scgb3a1*	secretoglobin, family 3A, member 1	NM_170727	ENSMUSG00000064057
*Scin*	scinderin	NM_009132	ENSMUSG00000002565
*Slc26a4*	solute carrier family 26, member 4	NM_011867	ENSMUSG00000020651
*Spdef*	SAM pointed domain containing ets transcription factor	NM_013891	ENSMUSG00000024215

### Immunohistochemistry and immunofluorescence

Formalin-fixed and paraffin embedded mouse lungs were cut into 2.5 μm sections for histological analysis. Sections were deparaffinized in xylene followed by decreasing concentrations of ethanol. Periodic acid–Schiff staining (PAS) and Sirius red staining was performed according to standard protocols. Antigen retrieval was performed using citrate buffer (pH6) at 95°C for 20 min. Primary antibodies against phospho-STAT6 (1:100; #9361S, Cell Signaling Technology, Boston, MA), smooth muscle actin (SMA; 1:30.000; A2547 Sigma Aldrich, St. Louis, MO) or von Willebrand factor (vWF; 1:10.000; A0082; DAKO/Agilent, Santa Clara, CA) were incubated for 1 h at room temperature. Primary antibodies were detected by the immPRESS α-Rabbit Ig (peroxidase) polymer detection kit using DAB Peroxidase (HRP) Substrate. Smooth muscle actin was detected using the Vector Vip Peroxidase (HRP) Substrate Kit (Vector Laboratories, Burlingame, CA). Counterstaining was performed for 10 s in 0.05% methylgreen in 0.1 M sodium acetat trihydrate (Sigma Aldrich, St. Louis, MO). Images were obtained using an Olympus VS120 slide scanning microscope at 40x magnification.

For double immunofluorescence, sections were incubated with antibodies against MUC5AC (1:100; ab212636) and CLCA1 (1:1000; ab180851, Abcam, Cambridge, UK), followed by incubation with Alexa Fluor 488 and 555 labelled secondary antibodies (1:500, Life Technologies, Carlsbad, CA). Slides were mounted with Vectashield DAPI containing mounting medium (Vector Laboratories, Burlingame, CA). Negative controls were performed alongside in each experiment by omission of the primary antibodies. Images were taken using a laser scanning confocal microscope (Nikon A1R Ultra-Fast Spectral Scanning Confocal Microscope) with a CFI Plan Apochromat Lambda 60x/1.4 oil immersion objective.

### Quantitative morphology and histology

The percentage of goblet cells and mucus volume was quantified on PAS stained sections using the NewCast software (Visiopharm, Hoersholm, Denmark) on automatically selected random regions from the 40x scanned images. 100 random regions were analysed per slide. Goblet and epithelial cells intersecting the airway basement membrane were counted and presented as percentage goblet cells per mm basement membrane; the volume of mucus was determined by point counts and compared to the surface area of the airway basement membrane as determined by line probe intersections (35, 36). The deposition of peribronchial collagen and smooth muscle thickness was analysed on Sirius red or α-SMA stained sections, respectively, using the Visiopharm image analysis software (VIS). For each animal 13 ± 7.8 bronchi, between 50 and 300 μm in size were analysed per slide. For both protocols, bronchi were outlined and collagen or smooth muscle staining was marked by automated colour recognition. Stained areas were skeletonized and the width of peribronchial collagen or the bronchial smooth muscle layer was calculated as follows: Area of staining/(interface length/2).

### Western blotting

Total proteins were isolated from lung homogenate using RIPA buffer (Sigma) and separated on a 10–12% SDS polyacrylamide gel and transferred to a PVDF membrane (GE Healthcare, Vienna, Austria). After blocking with 2.5% BSA in TBS-Tween (0.1%) buffer, the membrane was incubated overnight at 4°C with the following antibodies (diluted in 2.5% BSA): phospho-STAT6 (1:1000, #9361S, Cell Signaling), STAT6 (1:1000; #621, Santa Cruz, Dallas, TX) or anti-α-tubulin (1:5000; Cell Signaling). Horse radish peroxidase-conjugated goat anti-rabbit secondary antibodies together with the ECL prime (GE Healthcare, Little Chalfont, UK) developing solution was used to detect primary antibodies. Equal protein loading and transfer was controlled by normalisation to α-tubulin. Number of replicates is stated in each figure legend. Uncropped images of Western blot membranes are depicted in the Supplemental Material (Figures E2–E6).

### Bronchoalveolar lavage fluid (BALF)

After sacrifice animals were lavaged with 1 ml PBS containing the Pierce protease inhibitor cocktail (ThermoFisher Scientific) and 1 mM EDTA and total cell counts were made.

### Single cell lung tissue homogenates

Single cell lung tissue homogenates were performed as previously described (37). In short, the lower right lobe was cut into approximately 1 mm pieces and digested with 0.7 mg/ml Collagenase and 30 μg/ml DNAse in RPMI medium supplemented with 10% FCS, 2 mM glutamine and 1% penicillin-streptomycin (Gibco—ThermoFisher Scientific) for 40 min at 37°C. The tissue was passed through a 100 μm cell strainer to obtain a single cell suspension.

### Flow cytometry

BAL and single cell lung tissue homogenates were analysed using a LSRII flow cytometer and analysed with the FACSDiva software (BD Biosciences) as previously described (37). Cells were initially gated on CD45 positivity and were identified as follows: neutrophils (CD11b+, CD11c−, Gr-1+), macrophages (CD11b low, CD11c+, Siglec-F+), dendritic cells (CD11b+, CD11c+, MHC-II high), T helper cells (CD3+, CD4+), cytotoxic T cells (CD3+, CD8+), B cells (CD19+), and eosinophils (CD11b+, CD11c−, Siglec F+). Antibody details are provided in Table 2.

**Table 2:** Antibody details for flow cytometry.

**Antigen**	**Label**	**Company**	**Clone**	**Isotype**	**Dilution factor**
CD3	FITC	eBioscience	145-2C11	Hamster IgG	1:20
CD4	APC	Biolegend	GK1.5	Rat IgG2b, κ	1:100
CD8	PE	Biolegend	53-6.7	Rat IgG2a, κ	1:200
CD11b	V500	BD Bioscience	M1/70	Rat IgG2b, κ	1:50
CD11c	ef450	eBioscience	N418	Hamster IgG	1:50
CD19	AF700	Biolegend	6D5	Rat IgG2a, κ	1:100
CD24	PerCP Cy5.5	BD Bioscience	M1/69	Rat IgG2b, κ	1:500
CD25	APC-Cy7	Biolegend	PC61	Rat IgG1, λ	1:50
CD45	PerCP-Cy5.5	eBioscience	30-F11	Rat IgG2b, κ	1:200
CD45	FITC	Biolegend	30-F11	Rat IgG2b, κ	1:200
CD64	AF647	BD Bioscience	X54-5/7.1	Mouse IgG1, κ	1:20
gdTCR	BV421	Biolegend	GL3	Hamster IgG	1:50
Gr-1	PE-Cy7	Biolegend	RB6-8C5	Rat IgG2b, κ	1:800
MHC-II	APC-Cy7	Biolegend	M5/114.15.2	Rat IgG2b, κ	1:400
Siglec F	PE	BD Bioscience	E50-2440	Rat IgG2a, κ	1:20

### Cytokine assay

Cytokine concentrations in BAL fluid, lung homogenates (isolated in NP-40 buffer) and plasma of Fra2 TG and WT mice were measured via FlowCytomix Multiple Analyte Detection or Enzyme-linked immunosorbent assay (ELISA), (ThemoFisher Scientific). The experiments were performed according to manufacturers' protocol and cytokine concentrations were measured on a LSRII Flow Cytometer (BD Biosciences, USA) or using a SpectraMax Plus 384 spectrophotometer for ELISA, respectively.

### RNA isolation and real time PCR analysis

Mouse total RNA was isolated from lung homogenate samples using a peqGOLD Total RNA Kit (Peqlab, Erlangen, Germany). cDNA synthesis and real-time PCR was performed as described previously (23). Briefly, total RNA was reverse transcribed using the iScript™ cDNA Synthesis kit (Bio-Rad Laboratories, Hercules, CA, USA), according to manufacturer's instructions. Real-time PCR was performed using a LightCycler® 480 System (Roche Applied Science, Vienna, Austria). The PCR reactions were set up using a QuantiFast® SYBR® Green PCR kit (Qiagen, Hilden, Germany) using the following protocol: 5 min at 95°C, (5 s at 95°C, 5 s at 60°C, and 10 s at 72°C) × 45. Melting curve analysis and gel electrophoresis was performed to confirm the specific amplification of the expected PCR products. *Hmbs* and *B2m* were used as the reference genes. The difference in threshold cycle (Ct) values for each target gene was calculated as follows: ΔCt = meanCt reference genes–Ct target gene. Primer sequences are given in Table 3.

**Table 3:** Primer sequences for real-time PCR.

**Gene symbol**	**Forward primer (5′-3′)**	**Reverse primer (5′-3′)**
*Clca1*	GCTATGAGGGCATCGTCATC	TATGGAGAGGCCTGAGTCACC
*Muc5ac*	TGCTTCTGTCCTGAGGGTATG	TGCTTCTGTCCTGAGGGTATG
*Muc5b*	GAAACTGGAGCTGGGCTCTGG	CTCCGGTGAGTTCTAGATGTTCTG
*IL-4*	ATGGATGTGCCAAACGTCCT	TGCAGCTCCATGAGAACACT
*IL-5*	AAGCAATGAGACGATGAGGCT	CCCCACGGACAGTTTGATTCT
*IL-13*	GCCAAGATCTGTGTCTCTCCC	CCAGGTCCACACTCCATACC
*IFNg*	CAGCAACAGCAAGGCGAAAAAGG	TTTCCGCTTCCTGAGGCTGGAT
*IL-6*	ACAACCACGGCCTTCCCTACTT	CACGATTTCCCAGAGAACATGTG
*IL-17*	AGGACGCGCAAACATGAGTC	GGACACGCTGAGCTTTGAGG
*Agr2*	TTGGACGAATGCCCACACAG	GGACATACTGGCCATCAGGAG
*Scin*	ACACGCCTTTTTCAAGTCCG	AAGCCATTGTTTCGTGGCAG
*Adra2*	GGGACAGAAAACGCATCGTG	TAGGTCCGGGGTTTCAGAGT
*Ccl8*	GGAAGCTGTGGTTTTCCAGA	AGGTGTGAAGGTTCAAGGCTG
*RelmA*	TGGCTTTGCCTGTGGATCTT	GCAGTGGTCCAGTCAACGAGTA
*Ccl7*	CTGCTTTCAGCATCCAAGTGTG	CTTCCCAGGGACACCGACTAC
*Ccl11 (Eotaxin-1)*	ACAGCGCTTCTATTCCTGCTG	GCTCTTCAGTAGTGTGTTGGG
*Ccl24 (Eotaxin-2)*	AGTGGTTAGCTACCAGTTGGC	TGTATGTGCCTCTGAACCCAC
*Tff2*	GAAAGAACTGTGGCTTCCCG	CCATGACACACTGCTCCGAT
*Col6a5*	GGAAGGGACAACTGTGGACT	GAGGTGGGAAAGGGAACTGTC
*Mmp12*	TGATGCAGCTGTCTTTGACC	GTGGAAATCAGCTTGGGGTA
*Spdef*	GACGGACGACTCTTCTGACA	CTGTTCGTGGTGCCACATCT

### Assessment of airway hyperresponsiveness

Mice were anesthetized (150 mg/kg ketamine, 20 mg/kg xylazine), intubated, and mechanically ventilated (150 breaths/min, tidal volume of 10 ml/kg and a positive end expiratory pressure of two cmH2O) for the measurements of airway resistance, compliance and elastance using a FlexiVent (SciReq, Inc., Montreal, PQ, Canada) as previously described (37). Changes in airway lung function were calculated as response to increasing concentrations of methacholine (0, 1, 3, 10, 30 and 100 mg/ml, Sigma Aldrich). Each data point represents the average of twelve snapshot perturbations recorded over a 3-min period after each methacholine dose; before each set of perturbations two deep inflation manoeuvres were performed to normalise lung volume.

### Isolation and proliferation analysis of mouse tracheal smooth muscle cells

For the isolation of mouse airway smooth muscle cells, the trachea of 8 week old TG or WT mice was isolated. The surrounding adipose and connective tissue was carefully removed and the trachea was cut into 2–3 mm^2^ tissue pieces and put into 6-well culture plates. The tissue pieces were kept in DMEM-F12 culture medium supplemented with 10% FCS, 1% glutamine and antibiotics/antimycotics (ThermoFisher Scientific) until outgrowth of smooth muscle cells. After 1–2 weeks, cells were detached using Trypsin and put through a 100 μm cell strainer to remove tissue pieces before reseeding. Quality of smooth muscle cell isolation and purity was confirmed by IF staining of smooth muscle cell, fibroblast and epithelial cell markers. To measure the proliferation, 5,000 airway smooth muscle cells per well were seeded in a 96-well plate. After attachment cells were starved overnight in basal medium (DMEM-F12 supplemented with 0.2% penicillin/streptomycin) before measuring proliferation. Cells were incubated with basal medium supplemented with ^3^H-labelled thymidine (1 μCi1/ml, BIOTREND Chemikalien, Germany) and cell proliferation was determined after 24 h. For that, cells were harvested onto a 96-well filter plate. 25 μl Beta Plate scintillation cocktail (Perkin Elmer, Waltham, MA) were added to each well of the dried filter plate and radioactivity was counted on a Microbeta Trilux (PerkinElmer).

### Statistics

Statistical analysis was performed in R or Graphpad Prism using *t*-test for comparison of two groups or One-way analysis of variance (ANOVA) for the comparison of multiple groups with Bonferroni's *post-hoc* test to compare selected pairs of columns. For the comparison of dose–response curves a two-way ANOVA was used with Bonferroni's *post hoc* test. A *p* value ≤ 0.05 was considered statistically significant. Data are shown as scatterplots with a boxplot overlay showing the median and interquartile range, unless otherwise indicated.

## Results

### Asthma related and mucus production genes are regulated in Fra2 TG mice

**Figure 1:**
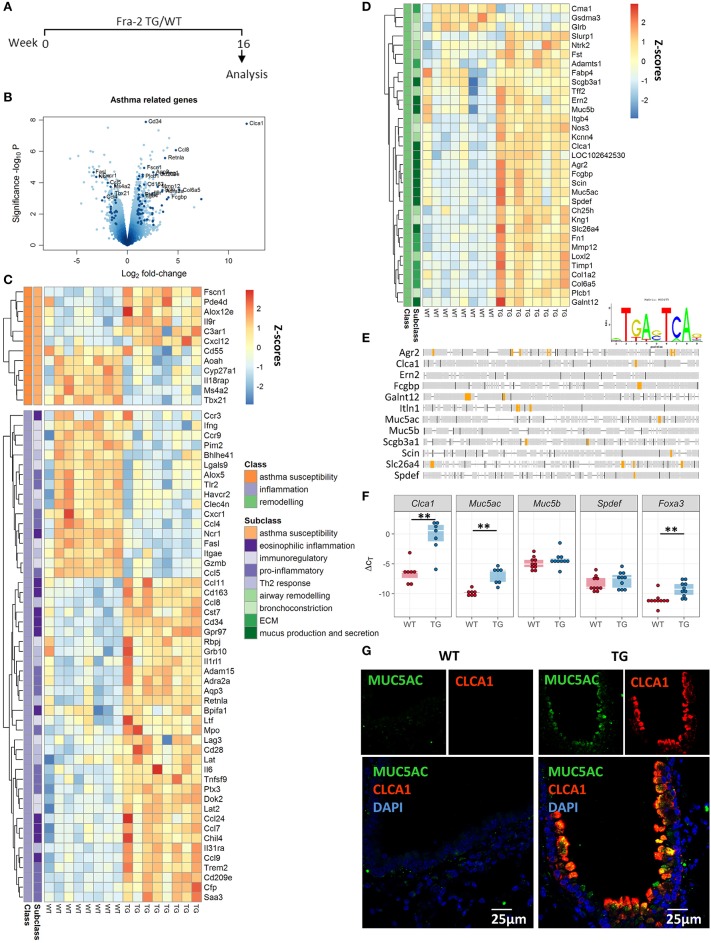
Asthma-related genes are differentially regulated in Fra2 TG mice. **(A)** Timeline of analysis of Fra2 overexpressing (transgenic/TG) and wild-type (WT) littermate control mice used in this study, *n* = 8 per group. **(B)** Volcano plot showing differential gene expression in the lungs of WT and TG mice, dark points indicate asthma-related genes. Labelled points represent asthma-related genes with Log_2_ fold-change > 1.5 and significance –log_10_*P* > 3. Heatmap representation of genes associated with **(C)** asthma susceptibility (orange), inflammation (purple) and **(D)** remodeling (green); Z-scores are shown. **(E)** AP-1 consensus sequence and *in silico* transcription factor binding site analysis for putative AP-1 binding sites (orange) in the proximal promoter region of genes involved in mucus production and secretion. **(F)** Quantitative real-time PCR analysis of *Clca1, Muc5ac, Muc5b, Spdef*, and *Foxa3* expression; *n* = 7–10; lines show median, ^**^p < 0.01. **(G)** Representative immunofluorescence staining (from *n* = 3) against MUC5AC (green) and CLCA1 (red) in the bronchi of Fra2 TGand WT mice.

We previously observed that Fra2 overexpression in mice causes age-dependent lung remodelling and peribronchial inflammation (Figure E1) (31). To understand how Fra-2 gives rise to these pathological changes, we performed gene expression profiling in 16 week-old Fra2 TG mice and WT littermates (Figure 1A). Importantly, at this time point TG mice possess inflammatory cell recruitment but no signs of parenchymal remodelling (31). In total 775 genes were regulated (≥2-fold change, *p* < 0.05), with 433 upregulated and 342 downregulated. Two of the highest regulated genes were *Clca1* (chloride channel accessory 1) and *Retnla* (resistin like alpha, also known as *Relma*/*Fizz1*) (Figure 1B), both of which are implicated in asthma (7, 38). These results prompted us to screen for further genes related to asthma. Within all regulated genes, a high proportion of genes (~12%) were associated with asthma susceptibility, inflammatory response (such as Th2 or eosinophilic inflammation) or airway remodelling (e.g., extracellular matrix deposition, or mucus production and secretion) (Figures 1C,D). Most striking, 9 of the 15 highest regulated (asthma related) genes were associated with mucus production and secretion (Figure 1D). *In silico* screening of the proximal promoter regions for transcription factor binding sites revealed multiple putative AP-1 binding sites in the majority (83%) of these genes (Figure 1E), indicating that their regulation might be a direct consequence of Fra2 overexpression. Using an independent mouse cohort, we verified the differential expression of *Clca1* and the airway mucin gene *Muc5ac* in lung homogenate samples by quantitative PCR (Figure 1F). Increased levels of CLCA1 and MUC5AC were also observed on the protein level as shown by immunofluorescence staining. In Fra2 TG mice both MUC5AC and CLCA1 showed a distinct signal in bronchial epithelial cells, whereas almost no signal could be detected in wild-type mice (Figure 1G). Furthermore the expression of *Spdef* and *Foxa3*, two transcription factors that regulate goblet cell differentiation and mucus production, was enhanced in Fra2 TG mice (Figure 1F) (8).

### Fra2 TG mice possess an asthmatic phenotype

**Figure 2:**
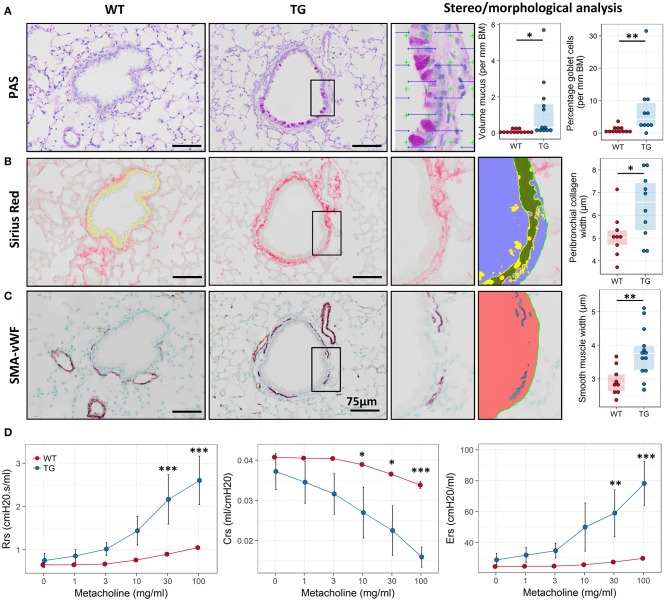
Fra2 TG mice exhibit airway remodeling and hyperresponsiveness. Representative images and subsequent stereological quantification of **(A)** Periodic acid-Schiff (PAS) staining for mucus volume and mucus producing goblet cells in the airways; **(B)** Sirius Red staining for peribronchial collagen and **(C)** double immunohistochemistry for von Willebrand-factor (vWF, brown) and α-smooth muscle actin (SMA, purple) for airway smooth muscle thickness. Insets show representative quantification. Points represent individual animals. **(D)** Analysis of lung function to determine changes in airway resistance (Rrs), compliance (Crs) and elastance (Ers) in response to increasing doses of methacholine in Fra2 TG (*n* = 5) and WT (*n* = 7) mice. ^*^p < 0.05, ^**^p < 0.01, ^***^p < 0.001.

Based on the expression profiling data we speculated that Fra2 overexpressing mice may exhibit an asthma-like phenotype and possess several of the hallmarks of asthma. Stereological analysis of PAS stained lung sections revealed pronounced numbers of goblet cells and mucus abundance in Fra2 TG mice (Figure 2A). Further remodelling was assessed by quantifying peribronchial collagen and smooth muscle layer thickness. Both peribronchial collagen as well as smooth muscle thickness was increased in TG mice (Figures 2B,C). We next determined if these structural changes manifest in altered lung function. Fra2 TG mice had an exaggerated methacholine response, with significantly increased resistance and elastance at higher concentrations and a concomitant decrease in dynamic compliance (Figure 2D).

### Increased inflammation in the BAL of Fra2 TG mice

**Figure 3:**
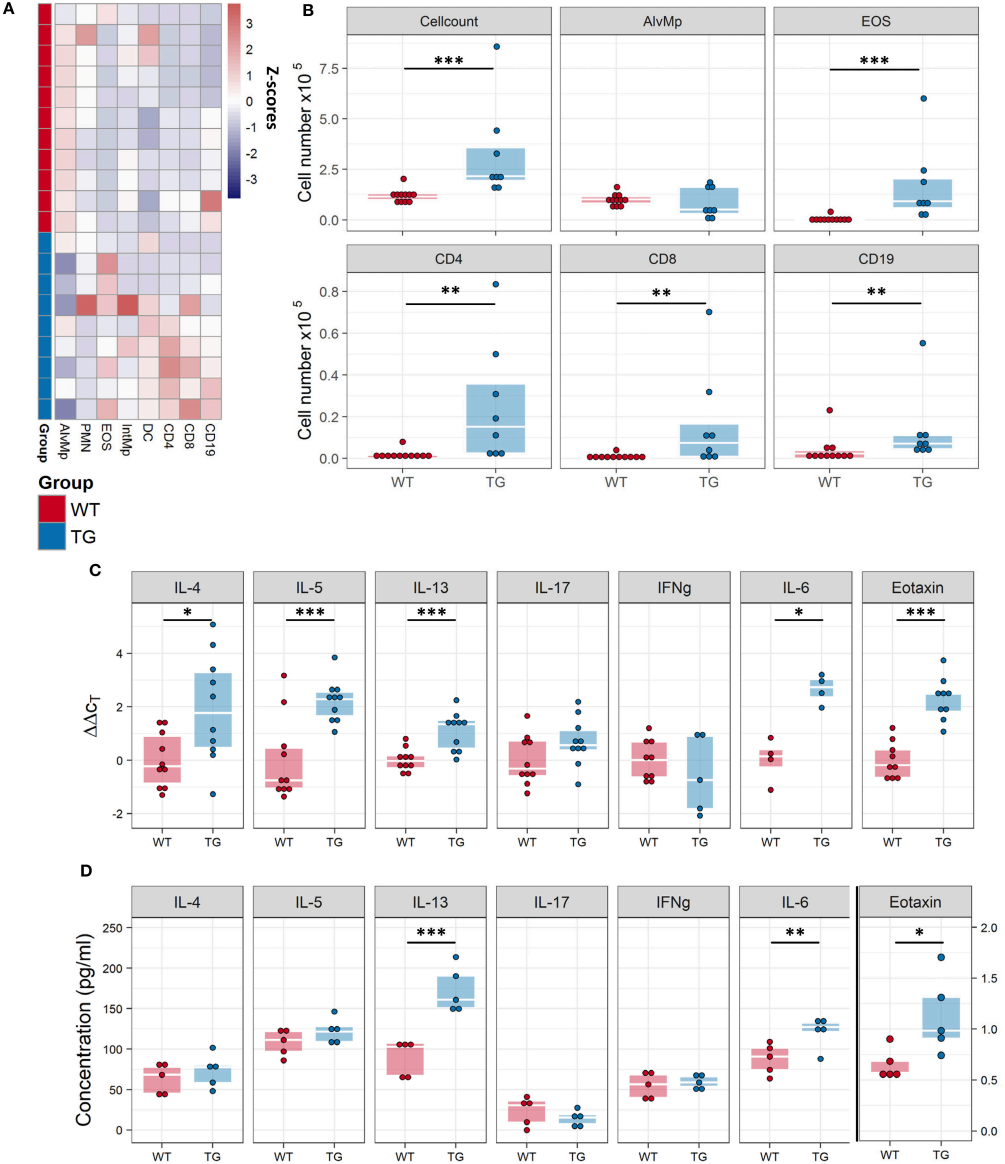
Fra2 TG mice possess increased airway inflammation. Flow cytometric analysis of inflammatory cells in the bronchoalveolar lavage fluid (BALF) of Fra2 TG mice and WT mice: **(A)** Heatmap overview of the relative proportion of inflammatory cell populations, Z-scores are shown. **(B)** Changes in the absolute abundance of individual cell populations. AlvMp, alveolar macrophages; PMN, polymorphonuclear neutrophils; EOS, eosinophils; IntMp, interstitial macrophages; DC, dendritic cells. *n* = 8 – 12 per group. **(C)** Quantitative real-time PCR analysis of mRNA levels and **(D)** protein levels of key inflammatory mediators in the lung homogenates of Fra2 TG mice and WT mice, note separate y-axis scale for Eotaxin protein concentrations. Points represent individual animals from two independent experiments. ^*^p < 0.05, ^**^p < 0.01, ^***^p < 0.001.

Consistent with the frequent inflammatory infiltration in the lungs of Fra2 TG mice (Figure E1), the total number of inflammatory cells in the BAL of TG mice was elevated. Analysis of the cells by flow cytometry revealed changes in the relative proportions of inflammatory cells (Figure 3A) and eosinophils as the most abundantly increased cell population (Figure 3B). Furthermore, from lymphoid populations both B-cells and T-cells (CD8^+^ effector as well as CD4^+^ T helper cells) were elevated. Analysis of cytokines levels revealed an increase in IL-6 and the Th2-cytokines IL-4, IL-5, and IL-13 on both mRNA and protein levels in Fra2 TG mice (Figures 3C,D). Furthermore, expression of eotaxin, a chemotactic factor for eosinophils was markedly increased (Figures 3C,D), which could contribute to the high abundance of eosinophils in Fra2 TG mice.

### IL-13 downstream signaling is increased in Fra2 TG mice

**Figure 4:**
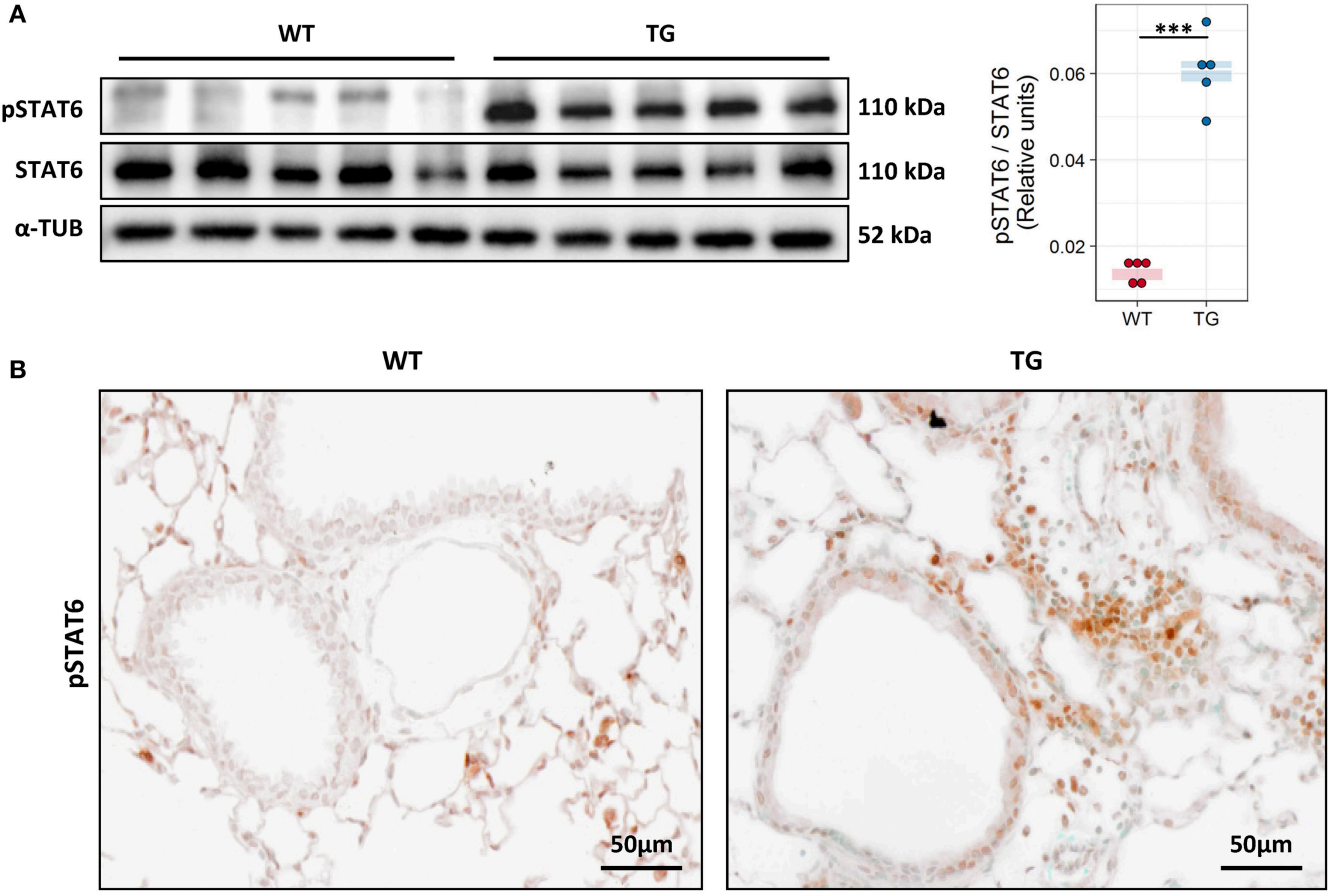
Fra2 TG mice possess strong activation of IL-13 downstream signaling. **(A)** Western blot analysis and quantification of phosphorylated (p) and total STAT6 levels in lung homogenates of Fra2 TG and WT mice (*n* = 5 per group); α-Tubulin (α-TUB) is shown as a loading control, ^***^p < 0.001. (Uncropped scans of the original membranes can be found in Figure E2) **(B)** Representative immunohistochemical staining (from *n* = 5) against pSTAT6 (brown).

Due to the raised levels of IL-13 and its importance in driving the allergic immune response we investigated the activation of its downstream signalling molecule signal transducer and activator of transcription 6 (STAT6). Analysis of STAT6 activation via Western blotting revealed high levels of phosphorylated STAT6 in TG mice (Figure 4A). Immunohistochemical staining against pSTAT6 revealed strong staining mostly in inflammatory cells around vessels and bronchi and in airway epithelial cells (Figure 4B).

### Blocking of IL-13 ameliorates airway hyperresponsiveness and eosinophilic cell recruitment

**Figure 5:**
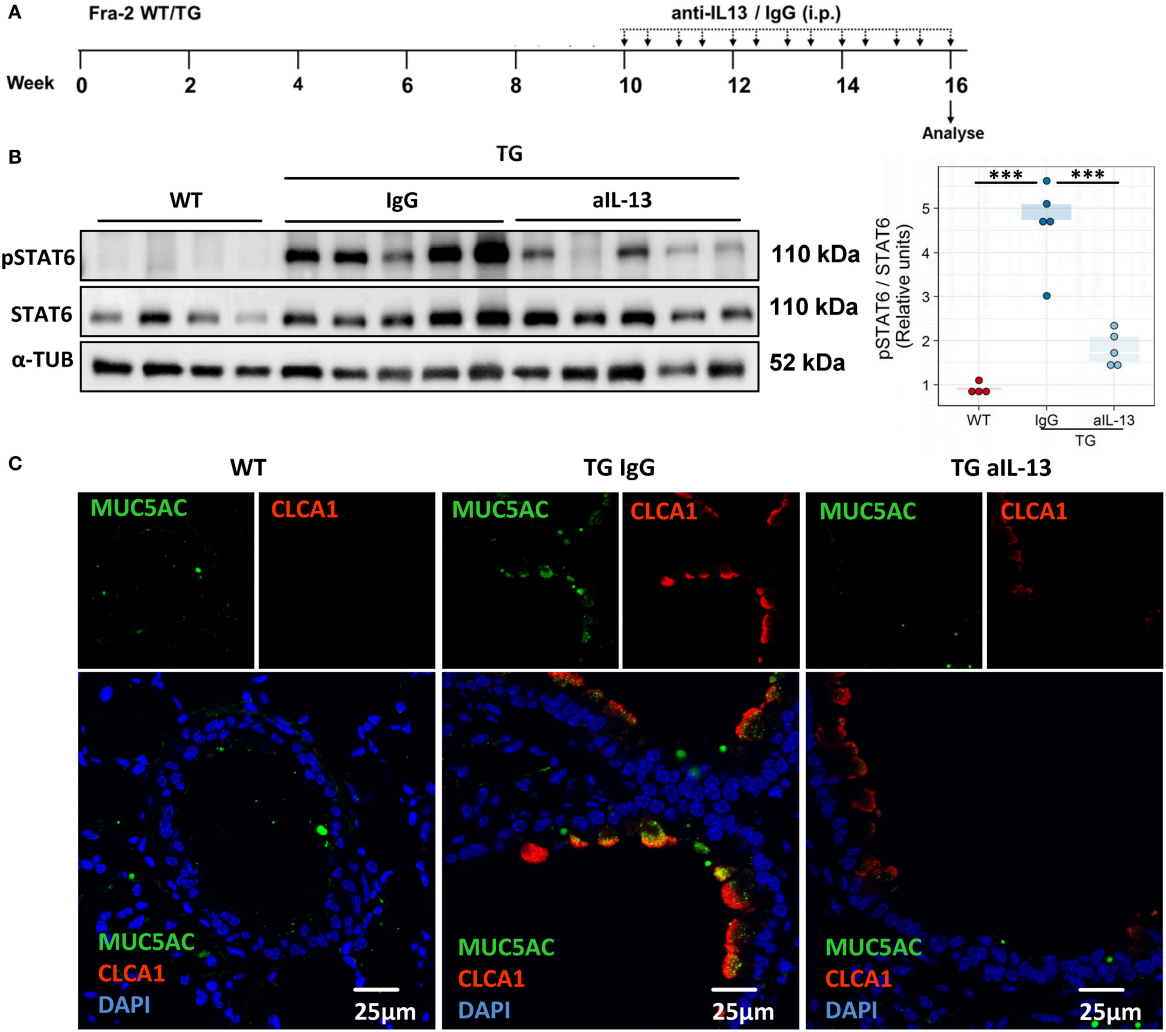
Blocking of IL-13 signaling decreases phosphorylation of STAT6 and mucus production in Fra2 TG mice. **(A)** Schematic representation of intraperitoneal (i.p.) treatment with IL-13 blocking antibody (anti-IL-13) or isotype control (IgG), *n* = 5 – 8 animals per group. **(B)** Western blot analysis and quantification of phosphorylated (p) and total STAT6 levels in mouse lung homogenates, ^***^p < 0.001. (Uncropped scans of the original membranes can be found in Figure E3). **(C)** Representative images (from *n* = 3) of immunofluorescence staining of MUC5AC (green) and CLCA1 (red) in bronchi of WT and Fra2 TG mice treated with control IgG or anti-IL-13 neutralising antibodies.

**Figure 6:**
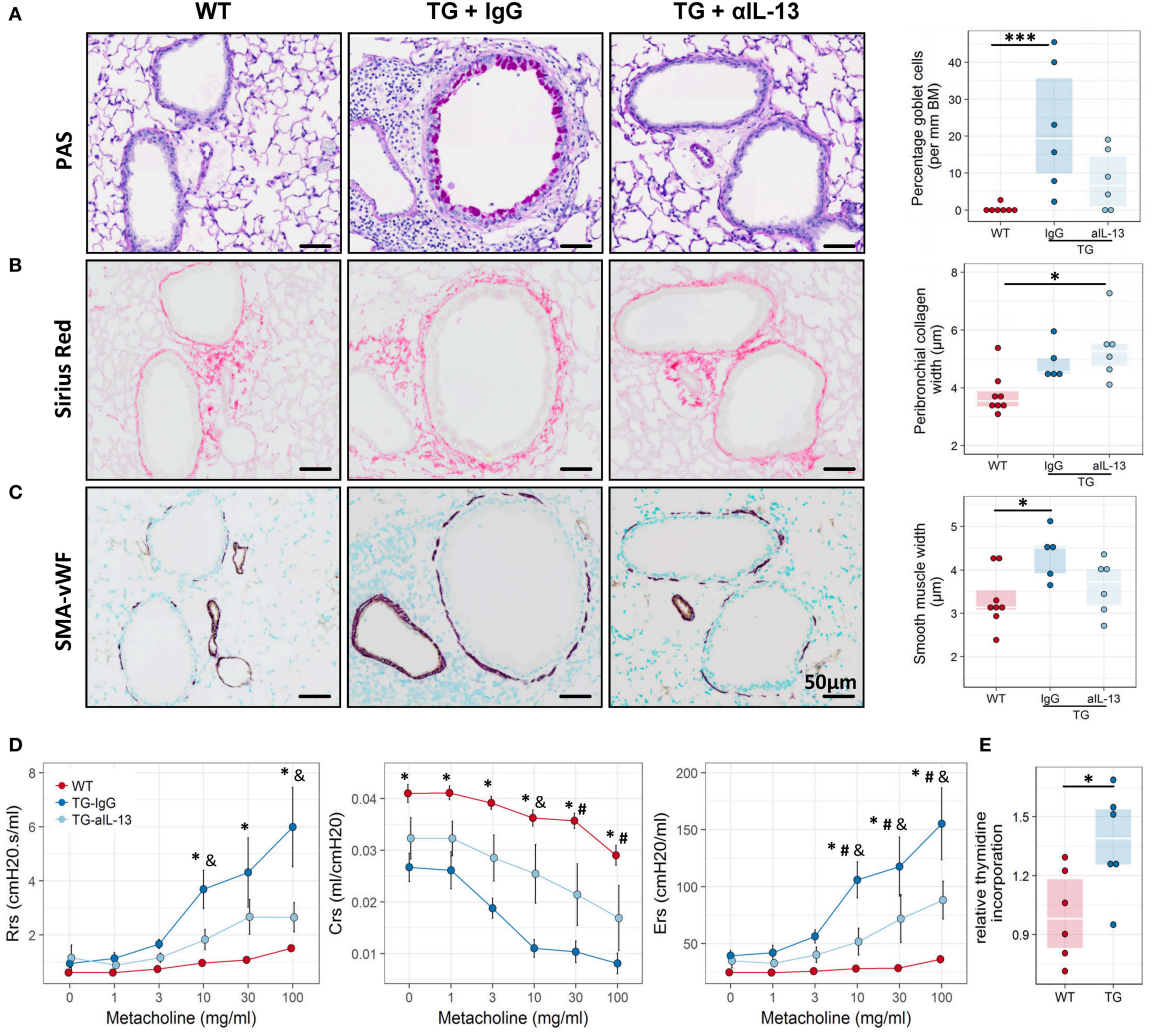
Blocking of IL-13 signaling ameliorates airway remodeling, hyperresponsiveness and inflammation in Fra2 TG mice. Representative images and subsequent quantification of **(A)** periodic acid-Schiff (PAS) staining for mucus producing goblet cells, **(B)** Sirius red staining for peribronchial collagen width and **(C)** double immunohistochemistry for von Willebrand-factor (vWF, brown) and α-smooth muscle actin (SMA, purple) for airway smooth muscle thickness. Points represent individual animals. ^*^p < 0.05, ^***^p < 0.001. **(D)** Lung function testing to determine changes in airway resistance (Rrs), compliance (Crs) and elastance (Ers) in response to increasing doses of methacholine in WT (*n* = 8) and Fra2 TG mice treated with isotype control (IgG, *n* = 5) or anti-IL-13 antibodies (*n* = 6). ^*^p < 0.05 WT vs. TG-IgG, # *p* < 0.05 WT vs. TG-aIL-13, &*p* < 0.05 TG-IgG vs. TG-aIL-13. **(E)** Proliferation of airway smooth muscle cells isolated from WT and Fra2 TG mice as determined by relative thymidine incorporation. Lines indicate the median, ^*^p < 0.05.

To assess whether IL-13 is the key driver of the asthmatic phenotype in Fra2 TG mice, we targeted IL-13 signalling via an IL-13 neutralising antibody (32) (Figure 5A). Blocking of IL-13 markedly decreased STAT6 phosphorylation in the lung homogenate samples as shown by western blot analysis (Figure 5B). Furthermore, CLCA1 and MUC5AC immunoreactivity was strongly decreased in TG mice after IL-13 blocking compared to untreated Fra2 TG mice (Figure 5C), which corresponded to a reduction in goblet cell hyperplasia (Figure 6A). However, peribronchial collagen deposition was similar between the two TG groups (Figure 6B). IL-13 blocking also resulted in a non-significant decrease in bronchial smooth muscle width in TG mice (Figure 6C). To assess whether increased airway remodelling could also be a direct effect of Fra2 overexpression, we isolated airway smooth muscle cells (ASMC) from wild-type and Fra2 TG mice and assessed proliferation. Overexpression of Fra2 increased the basal proliferation of ASMCs (Figure 6E), which indicates that airway smooth muscle thickening can also be a direct effect of the ectopic Fra2 overexpression. Analysis of lung function parameters revealed decreased AHR in mice treated with IL-13 blocking antibody (Figure 6D).

**Figure 7:**
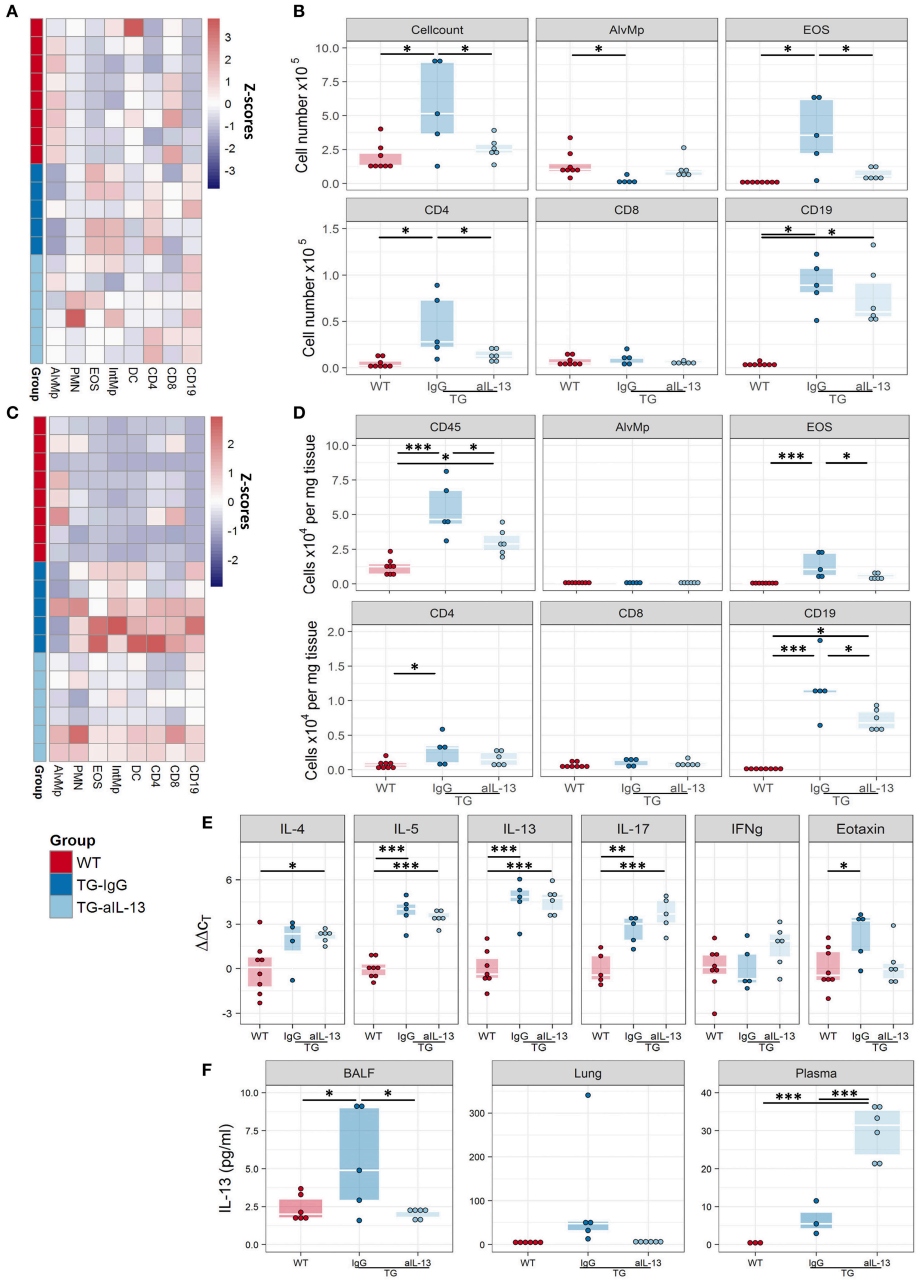
Blocking of IL-13 signaling decreases inflammation in Fra2 TG mice. Flow cytometric analysis of inflammatory cells in the bronchoalveolar lavage fluid **(A,B)** and of lung homogenates **(C,D)** of WT (*n* = 8) or Fra2 TG mice treated with anti-IL-13 neutralising (*n* = 6) or isotype (IgG, *n* = 5) antibodies: (**A,C**) Heatmap overview of the relative proportion of inflammatory cell populations, Z-scores are shown. **(B,D)** Changes in the absolute abundance of individual cell populations. AlvMp, alveolar macrophages; PMN, polymorphonuclear neutrophils; EOS, eosinophils; IntMp, interstitial macrophages; DC, dendritic cells. **(E)** Quantitative real-time PCR analysis of key inflammatory mediators in mouse lung homogenates. **(F)** IL-13 protein levels measured in lung homogenate (LH), bronchoalveolar lavage fluid (BALF) and plasma of WT and Fra2 TG mice treated with isotype control (IgG) or anti-IL-13 antibodies. ^*^p < 0.05, ^**^p < 0.01, ^***^ *p* < 0.001.

Blocking of IL-13 signalling altered the inflammatory profile in the BALF of Fra2 TG mice (Figure 7A) and diminished the number of total inflammatory cells, eosinophils and T-cells (Figure 7B). A similar pattern of inflammatory cell recruitment could be observed in lung homogenates of WT mice and TG mice treated with IL-13 blocking or isotype control antibodies (Figures 7C,D). IL-4, IL-5, and IL-13 levels were unaltered in the lung homogenates of treated Fra2 TG mice compared to untreated mice, demonstrating that IL-4 and IL-5 expression is not downstream of IL-13 in this mouse model. The expression of eotaxin decreased, whereas IL-17 production increased following IL-13 inhibition (Figure 7E). Increased IL-13 protein was also observed in TG compared to WT mice; treatment with IL-13 blocking antibodies strongly decreased IL-13 levels in BALF and lung samples (Figure 7F). In contrast, treatment significantly increased circulating IL-13 in the plasma of TG mice (Figure 7F).

### Steroid-treatment partially ameliorates the Fra2 TG phenotype

**Figure 8:**
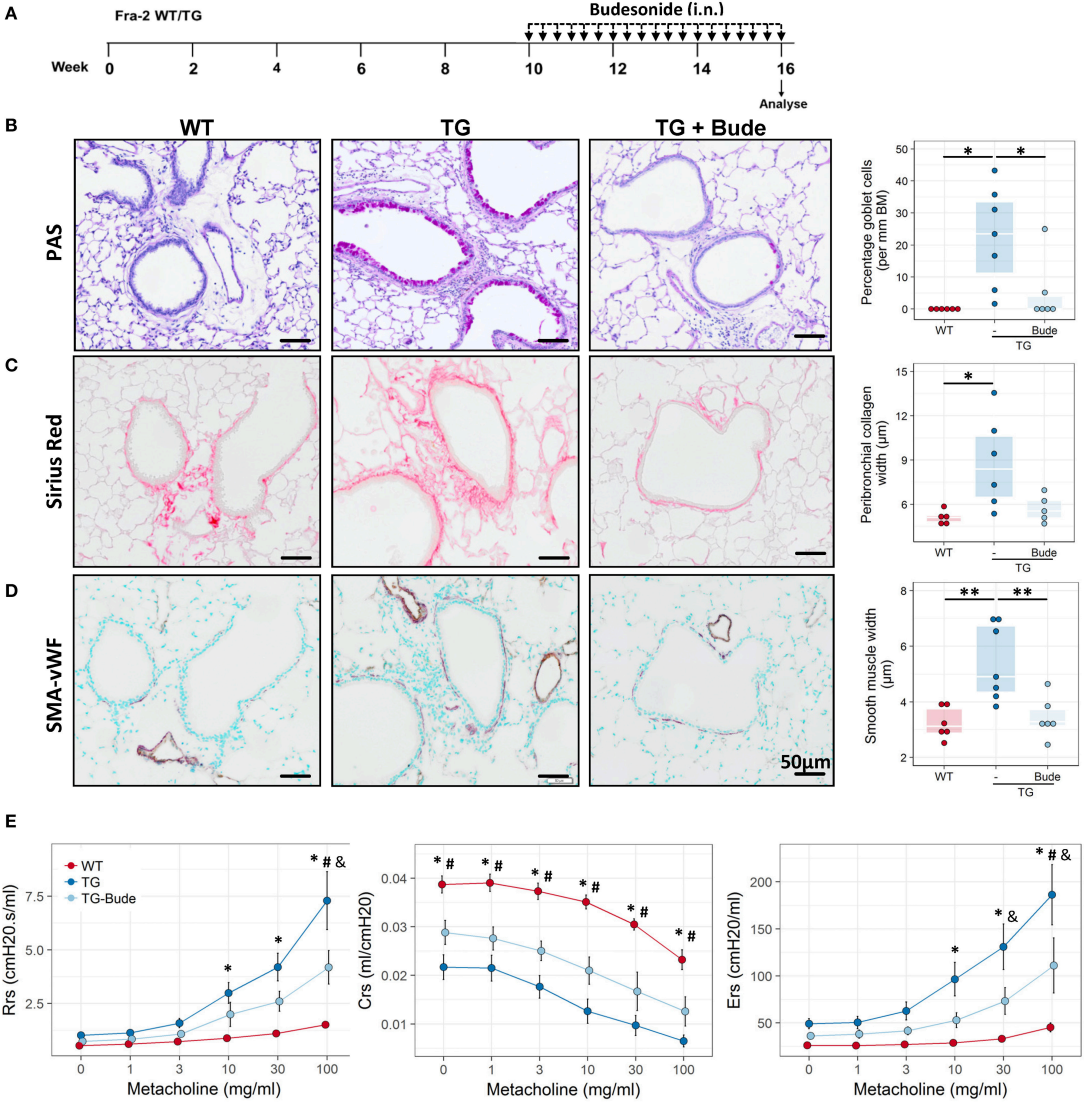
Glucocorticoid treatment partially attenuates the Fra2 TG phenotype. **(A)** Schematic representation of intranasal (i.n.) budesonide treatment in Fra2 TG mice (*n* = 6 – 7 animals per group). Representative images and quantification of **(B)** periodic acid-Schiff (PAS) staining for mucus producing goblet cells, **(C)** Sirius red staining for peribronchial collagen width and **(D)** double immunohistochemistry for von Willebrand-factor (vWF, brown) and α-smooth muscle actin (SMA, purple) for airway smooth muscle thickness. Budesonide (Bude), data points represent individual animals. ^*^p < 0.05, ^**^p < 0.01. **(E)** Lung function testing to determine changes in airway resistance (Rrs), compliance (Crs) and elastance (Ers) in response to increasing doses of methacholine in WT and Fra2 TG mice with or without budesonide (Bude) treatment; *n* = 6 per group. ^*^p < 0.05 WT vs. TG, #*p* < 0.05 WT vs. TG-Bude, & *p* < 0.05 TG vs. TG-Bude.

**Figure 9:**
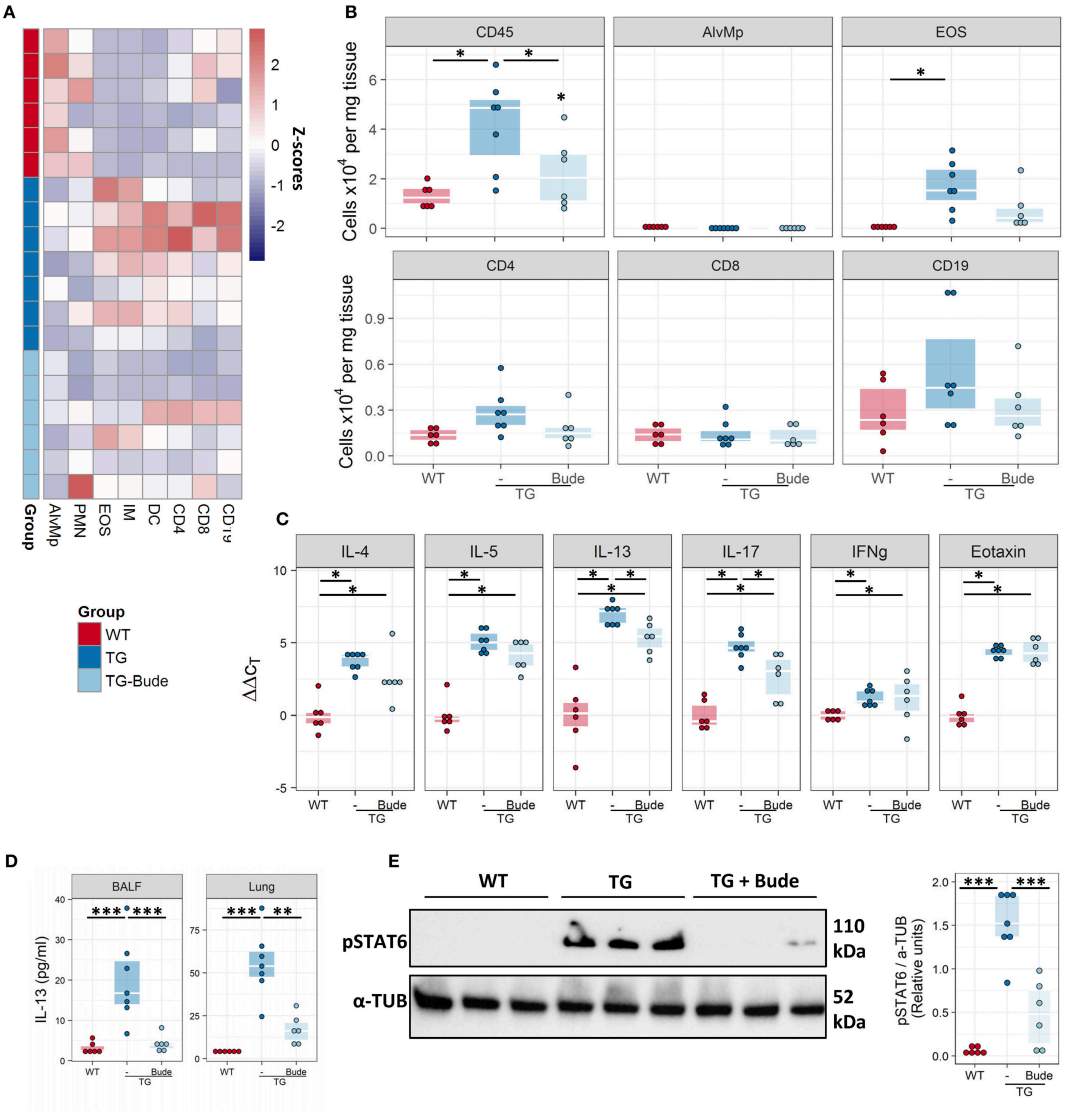
Budesonide decreases lung inflammation in Fra2 TG mice. Flow cytometric analysis of inflammatory cells in the lung homogenates of WT (*n* = 6) or Fra2 TG mice treated with (*n* = 7) or without Budesonide (*n* = 6): **(A)** Heatmap overview of the relative proportion of inflammatory cell populations, Z-scores are shown. **(B)** Changes in the absolute abundance of individual cell populations. AlvMp, alveolar macrophages; PMN, polymorphonuclear neutrophils; EOS, eosinophils; IM, interstitial macrophages; DC, dendritic cells. **(C)** Quantitative real-time PCR analysis of key inflammatory mediators in mouse lung homogenates. ^*^p < 0.05. **(D)** IL-13 protein levels measured in the bronchoalveolar lavage fluid (BALF) and lung homogenate (Lung) of WT and Fra2 TG mice treated with or without Budesonide. **(E)** Western blot analysis and quantification of phosphorylated (p) and total STAT6 levels in lung homogenates of WT and Fra2 TG mice with or without budesonide (Bude) treatment; one representative of two western blots is shown. α-Tubulin (α-TUB) is shown as a loading control. (Uncropped scans of the original membranes can be found in Figure E4). ^**^p < 0.01, ^***^p < 0.001.

It has been hypothesised that elevated levels of pro-inflammatory transcription factors such as AP-1 can lead to insensitivity to glucocorticoids (39). We therefore examined whether Fra2 overexpression gives rise to a steroid-refractory asthma phenotype. Treatment with the glucocorticoid budesonide (Figure 8A) reduced goblet cell hyperplasia, peribronchial collagen as well as airway smooth muscle thickness (Figures 8B–D). A concomitant partial improvement in lung function was observed, with budesonide treatment reducing AHR to a level between WT and TG mice (Figure 8E). Furthermore, budesonide decreased inflammatory cell recruitment, strongly reducing eosinophil numbers in the lungs (Figures 9A,B), along with a slight reduction of the expression of the Th2 cytokines (IL-4, IL-5, and IL-13; Figure 9C). Budesonide strongly decreased IL-13 protein levels in the BALF and lung homogenates (Figure 9D) and blocked IL-13 downstream signalling as indicated by the loss of STAT6 phosphorylation in TG mice following budesonide treatment (Figure 9E).

### Anti-inflammatory treatment reduces Fra2 protein levels in Fra2 TG mice

**Figure 10:**
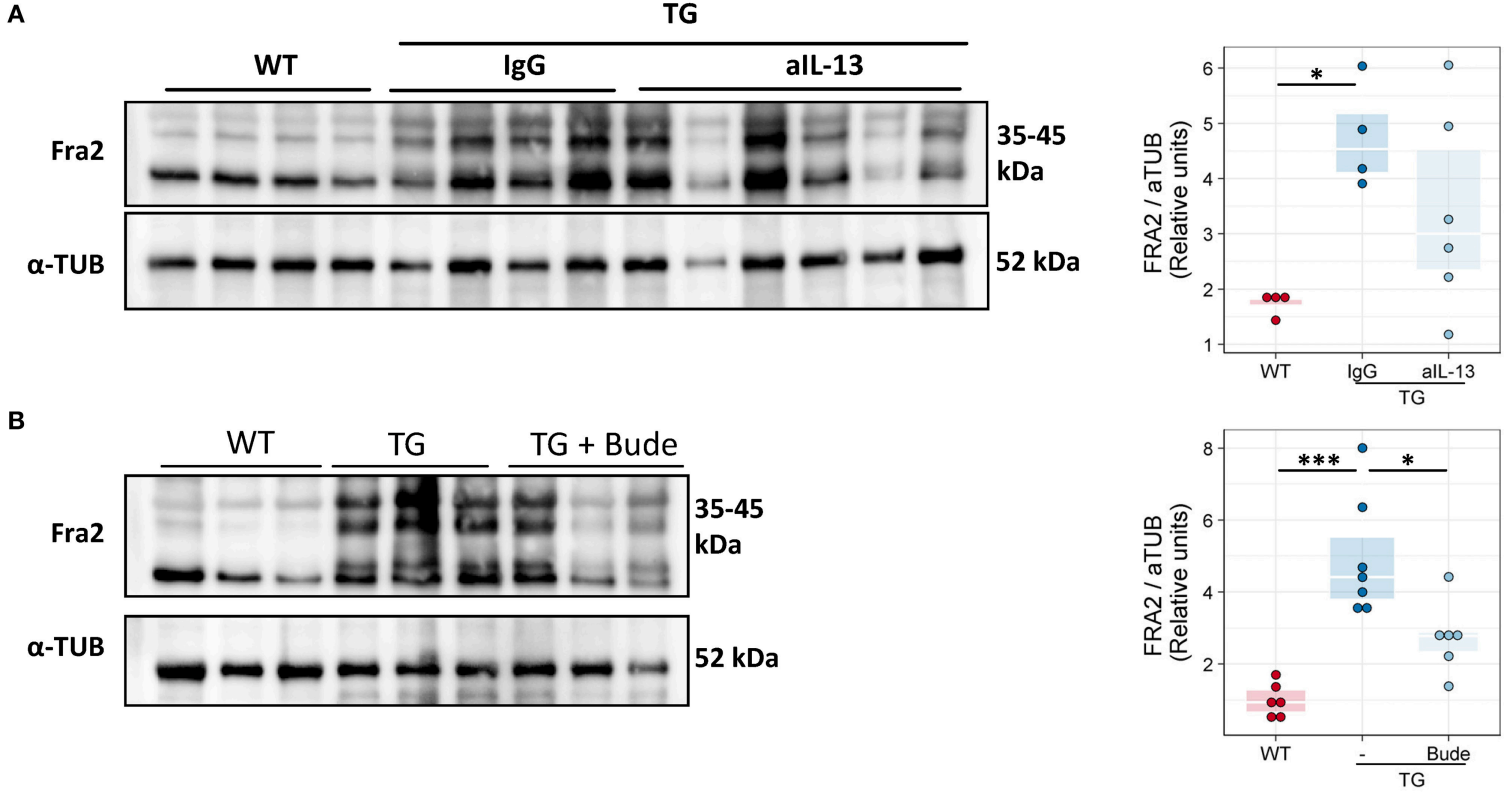
Fra2 protein levels decrease after anti-inflammatory treatment of Fra2 TG mice. Western blot analysis and quantification of Fra2 in the lungs of **(A)** WT and Fra2 TG mice treated with isotype control (IgG) or anti-IL-13 antibodies (aIL-13); or **(B)** WT and Fra2 TG mice treated with or without Budesonide; one representative of two western blots is shown. α-Tubulin (α-TUB) is shown as a loading control. (Uncropped scans of the original membranes can be found in Figures E5, E6); *n* = 5–8. ^*^p < 0.05, ^***^p < 0.001.

Finally we examined whether our intervention strategies could alter the abundance of the Fra2 protein. Glucocorticoid treatment led to decreased Fra2 protein levels in TG mice (Figure 10). Increased phosphorylation of Fra2 could be observed by the appearance of higher molecular weight bands (~40–45 kDa) in Fra2 TG mice. Neither treatment reduced Fra2 expression to levels comparable to WT mice (Figure 10).

## Discussion

Asthma is a complex chronic disease with symptoms such as wheezing, cough or dyspnoea. The underlying pathology includes airway remodelling, excessive mucus production, and inflammatory cell recruitment (2). Current animal models of asthma predominately rely on the sensitisation and challenge with exogenous allergens and mimic atopic asthma (12), however, there is a lack of pre-clinical models that represent other asthma phenotypes, such as non-atopic asthma. In this study we have conclusively shown that Fra2 overexpressing mice spontaneously develop a severe asthmatic phenotype without the need of additional allergen challenge. TG mice possessed several key features of asthma including airway inflammation with pronounced eosinophilia and Th2 inflammation, airway structural changes including mucus hypersecretion and AHR. Although some aspects of asthma in Fra2 TG mice could be ameliorated by blocking IL-13 signalling or by treatment with glucocorticoids, the phenotype was partially resistant to both treatments. This model may therefore be more reflective of late-onset asthma (non-allergic asthma), which often exhibits poor response to classical glucocorticoid treatment (3).

Although, AP-1 is an important component of multiple signalling cascades controlling cell differentiation and proliferation, little is known about which specific AP-1 subunits are involved in the development of asthma and studies investigating specific subunits are limited (28, 29). The composition of the AP-1 complex defines its affinity to DNA sequences around the core AP-1 site and ultimately determines which genes are regulated (40). Elevated c-fos levels have been described in an OVA-induced rat asthma model (41), and in bronchial biopsies from asthmatic patients (42). JunB has been shown to be important for the development of murine experimental asthma, due to its role in Th2 cell differentiation and the production of Th2 cytokines (43). We here show that the lesser known member of the AP-1 family, Fra2 is a common denominator in several aspects of asthmatic airway disease. Using an unbiased gene expression profiling approach, we showed that the Fra2 induced asthmatic phenotype is not only due to the pro-inflammatory actions of AP-1 leading to Th2/eosinophilic inflammation, but also to its involvement in the regulation of genes associated with airway remodelling and mucus hypersecretion.

Regulation of airway mucus production occurs on two separate levels; transcriptional control of MUC genes or via the increased secretion of stored mucin (44). Our study demonstrates that Fra2 regulates mucus hypersecretion at multiple stages. Overexpression of Fra2 increases the production of MUC5AC, one of the main mucins produced in the airways, and also upregulates expression of genes crucial for the correct hydration and secretion of mucus e.g. *Clca1* (45). Additionally Fra2 increases the expression of transcription factors, e.g., *Foxa3*, important for goblet cell differentiation (8). These changes translate into high numbers of mucus producing goblet cells and mucus production in the airways of Fra2 TG mice. TG mice also possessed increased peribronchial collagen deposition, which supports earlier observations that Fra2 drives the expression of *Col1a1* and *Col1a2* (46, 47). Further evidence linking Fra2 to structural changes and remodelling of airways is the increased smooth muscle thickness in TG mice and enhanced proliferation of airway smooth muscle cells overexpressing Fra2.

Although increased inflammation and IL-4 upregulation was originally described in Fra2 TG mice (30), follow-up studies focused more on the vascular manifestations and the development of fibrosis in the skin and lung in older mice (31, 48, 49). Here we conducted a thorough characterisation of the inflammatory infiltrates in the lungs of these mice and found predominant eosinophilia and Th2 inflammation with high levels of the effector cytokine IL-13. The efficacy of targeting IL-13 was observed on multiple levels; decreased IL-13 levels in the BALF and lung, loss of STAT6 phosphorylation, eotaxin production and attenuated eosinophil recruitment, supporting the essential nature of IL-13 in these processes (50, 51). Interestingly, functional blocking of IL-13 significantly increased circulating IL-13 in the plasma, possibly due to disturbed internalisation and clearing of IL-13 (52). Furthermore, IL-13 inhibition strongly decreased goblet cell hyperplasia and mucus production, but did not affect peribronchial collagen thickness. This further supports the direct influence of Fra2 on collagen production and suggests that some structural/morphological changes in this model are independent of IL-13 but Fra2 dependent. However, it is also possible that the treatment duration or dose was insufficient for complete restoration of the phenotype. Blocking of IL-13 led to an overall decrease of inflammation with significantly less eosinophils and T-cells, but increased neutrophil numbers and IL-17 levels. Anti-IL-13 therapy has previously been associated with exacerbated airway neutrophilia in the lungs of animals exposed to ovalbumin and ambient particulate matter (32). As IL-13 can negatively regulate IL-17 production by Th17 cells (53), blocking of IL-13 signalling might not only have beneficial effects, but lead to a shift in inflammation toward a Th17/neutrophil predominant inflammation as observed in our mice. These results highlight the importance to carefully choose suitable treatment strategies for distinct asthma endotypes, since such adverse effects might be detrimental for patients with mixed eosinophilic/neutrophilic asthma.

The Fra2 induced asthma phenotype could be partially ameliorated by treatment with the glucocorticoid budesonide. Budesonide exerted potent anti-inflammatory effects and decreased inflammatory cell recruitment in the lungs of TG mice. Budesonide was also able to decrease airway smooth muscle thickness. These beneficial effects could be explained in a number of ways: (1) Direct inhibition of AP-1 activity via direct interaction between the glucocorticoid receptor (GR) and AP-1/Fra2 (54). (2) Increased turnover of Fra2 protein, as observed by reduced Fra2 protein levels following budesonide treatment. (3) Blockade of AP-1 downstream effects by interacting with other pro-inflammatory transcription factors such as NF-κB or upregulation of anti-inflammatory genes (55). Abnormalities in the interaction of GR and AP-1 have been postulated to be one of the causes of steroid refractory asthma (56). Changes in the composition of the AP-1 complex due to overexpression of Fra2, may therefore decrease its binding affinity to the GR and consequently explain the incomplete attenuation of the Fra2 phenotype by budesonide and the steroid-insensitivity in some asthmatic individuals.

Anti-inflammatory treatment partially reduced Fra2 protein levels in the lungs of TG mice, indicating that endogenous expression of Fra2 due to pro-inflammatory stimuli, such as IL-13 (20) accounts for some of the Fra2 production. The constant presence of Fra2 (due to ectopic overexpression) triggering expression of pro-inflammatory Th2 cytokines might therefore lead to a vicious cycle, rendering normal resolution of inflammation impossible. AP-1 itself could therefore serve as a therapeutic target. In an OVA-induced asthma mouse model, inhibition of redox-regulated AP-1 transcriptional activity proved beneficial for the asthmatic phenotype and decreased inflammation (57). Similar results were obtained by using decoy nucleotides specifically blocking AP-1 DNA binding and transcription of target genes (29). Blocking AP-1 activity could therefore be a potential treatment strategy in asthmatic airway disease and might prove beneficial by interfering with several aspects of disease pathology (such as inflammation and airway remodelling). Furthermore, abnormal airway inflammatory responses typify asthma exacerbations which are a common response to viral infections or allergen exposure, leading to increased hospitalisation and higher health-care costs (58). The mechanisms underlying these exacerbations are not fully elucidated. Here the Fra2 TG mice could be a valuable tool to investigate allergen- or viral-induced asthma exacerbations.

In summary, we have shown that the AP-1 transcription factor family member Fra2 acts strongly pro-inflammatory, leading to a prominent Th2 driven inflammation in the lungs of mice, without the need for additional allergen challenge. Fra2 TG mice developed severe airway disease including airway remodelling and declined lung function with AHR. This phenotype could only partially be reversed by blocking IL-13 signalling or by anti-inflammatory treatment with glucocorticoids, suggesting that morphological and functional changes were due to a combination of direct Fra2 overexpression and IL-13 pathway activation. However, further investigations are needed to clarify the role of Fra2 in other animal models of experimental asthma and in human airway disease.

## Author contributions

AG, GK, LM conceived and designed the study. AG, VB, JW, LM were involved in the acquisition and analysis of data. AG and LM drafted and wrote the manuscript. All authors participated in the interpretation of results and critically revised the manuscript. All authors approved the final version of the manuscript.

### Conflict of interest statement

The authors declare that the research was conducted in the absence of any commercial or financial relationships that could be construed as a potential conflict of interest.

## Abbreviations

AHR: Airway hyperresponsiveness
Activator protein 1, AP-1, BALF: Bronchoalveolar lavage fluid
IL: interleukin
PBS: Phosphate buffered saline
qPCR: quantitative real-time polymerase chain reaction
TG: transgenic
WT: wild-type mouse.
