# Supplementary material for: Fra2 Overexpression in Mice Leads to Non-allergic Asthma Development in an IL-13 Dependent Manner
Source: Front Immunol. 2018 Sep 5;9:2018. doi: 10.3389/fimmu.2018.02018 (PMC6133984; doi:10.3389/fimmu.2018.02018)
Supplement: Supplementary file 1 [file Data_Sheet_1.pdf]

Supplementary Material

Fra2 Overexpression in Mice Leads to Non-allergic Asthma Development in an IL-13 Dependent Manner

Anna Gungl[1], Valentina Biasin[2], Jochen Wilhelm[3,4], Andrea Olschewski[1,2], Grazyna Kwapiszewska[1,2], Leigh M. Marsh[2*]

[1]Otto Loewi Research Center, Medical University of Graz, Graz, Austria

[2]Ludwig Boltzmann Institute for Lung Vascular Research, Graz, Austria

[3]Department of Internal Medicine, Universities of Giessen and Marburg Lung Center, Giessen, Germany

[4]German Center for Lung Research, Justus-Liebig University, Giessen, Germany

***Correspondence:**

Leigh M. Marsh

Ludwig Boltzmann Institute for Lung Vascular Research

Stiftingtalstrasse 24, 8010 Graz, Austria

E-mail: Leigh.Marsh@lvr.lbg.ac.at

Figure legends

Figure E1: Peribronchial inflammation in Fra2 TG mice.

Hematoxylin and eosin (HE) staining of airways in WT and Fra2 TG mice.

Figure E2:

Uncropped images of the Western blot membranes shown in Figure 4A. St: Standard; WT: wild-type; TG: Fra2 transgene.

Figure E3:

Uncropped images of the Western blot membranes shown in Figure 5B. St: Standard; WT: wild-type; TG: Fra2 transgene, IgG: isotype control; aIL-13: anti-IL-13 neutralising antibody.

Figure E4:

Uncropped images of the Western blot membranes shown in Figure 9E. St: Standard; WT: wild-type; TG: Fra2 transgene; Bude: Budesonide.

Figure E5:

Uncropped images of Western blot membranes shown in Figure 10A. St: Standard; WT: wild-type; TG: Fra2 transgene, IgG: isotype control; aIL-13: anti-IL-13 neutralising antibody.

Figure E6:

Uncropped images of the Western blot membranes shown in Figure 10B. St: Standard; WT: wild-type; TG: Fra2 transgene; Bude: Budesonide.

Figure E1

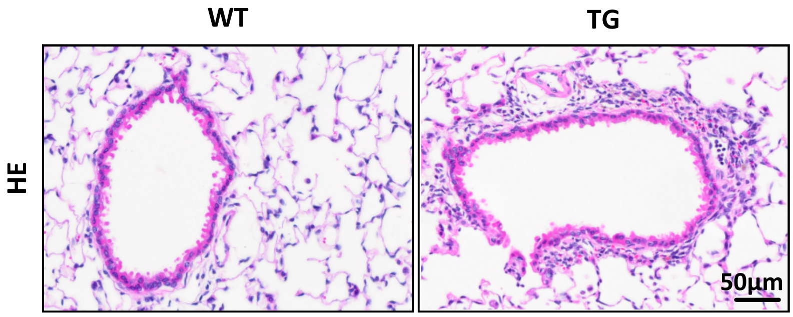

Figure E2

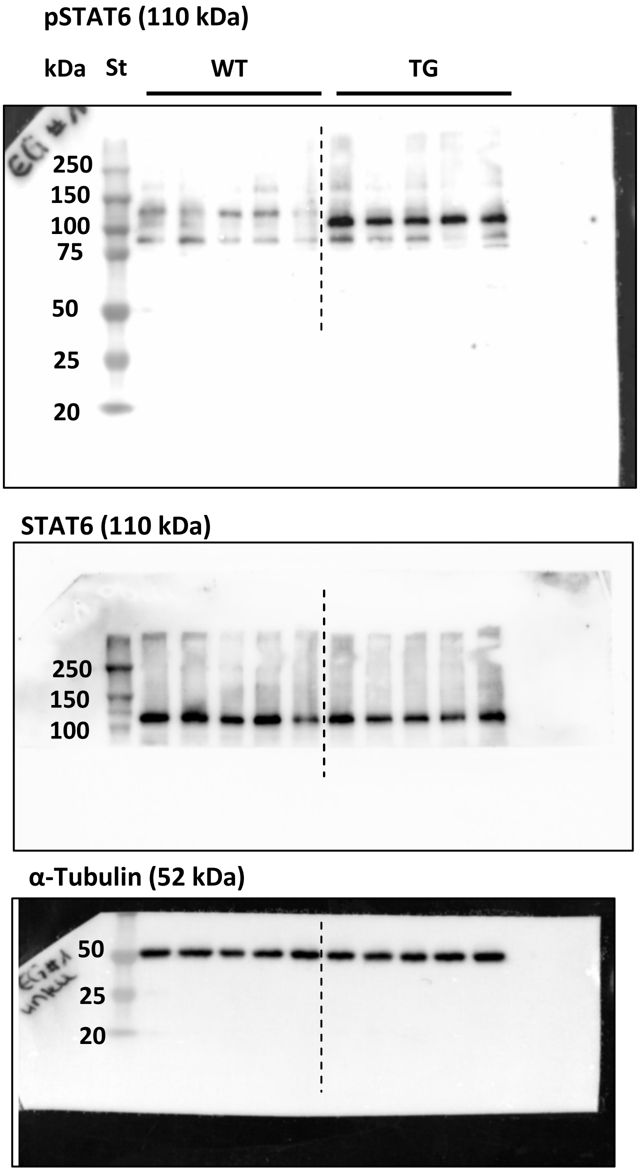

(Figure 4A)

Figure E3

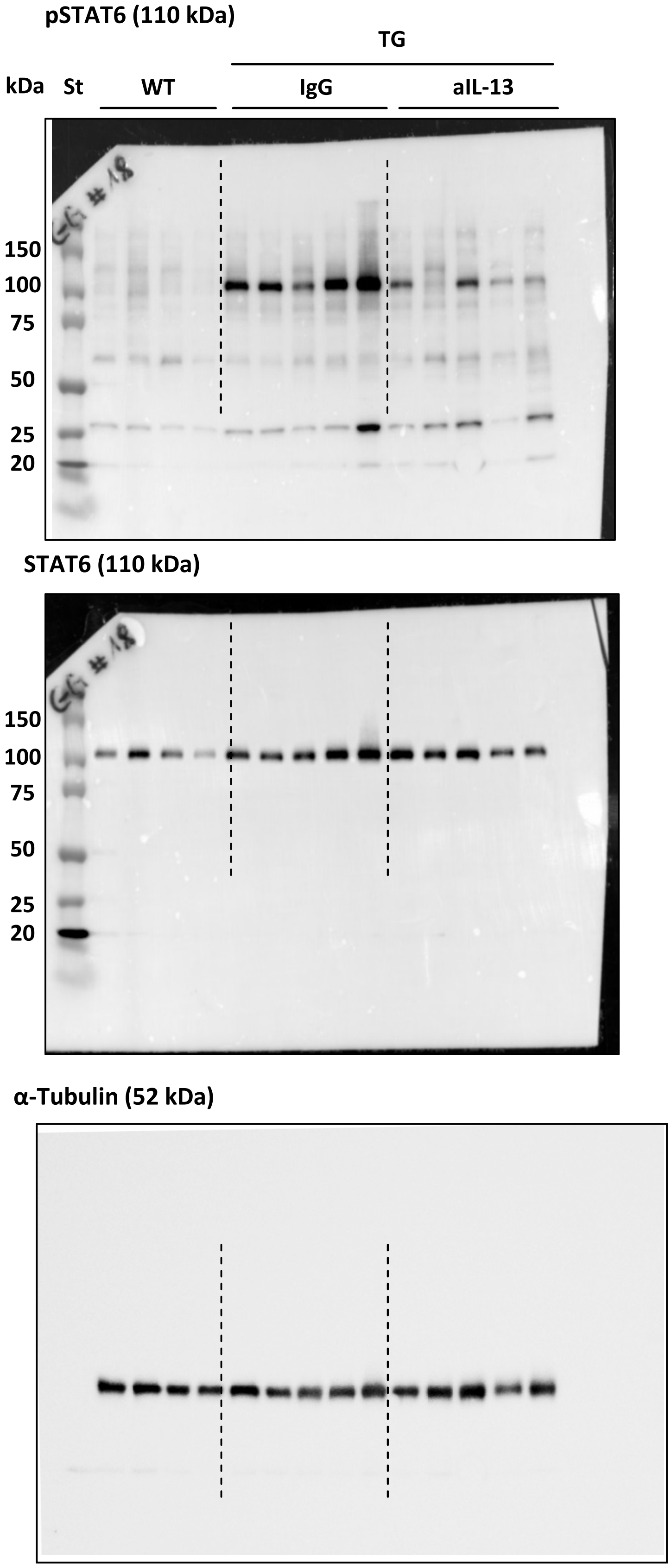

(Figure 5B)

Figure E4

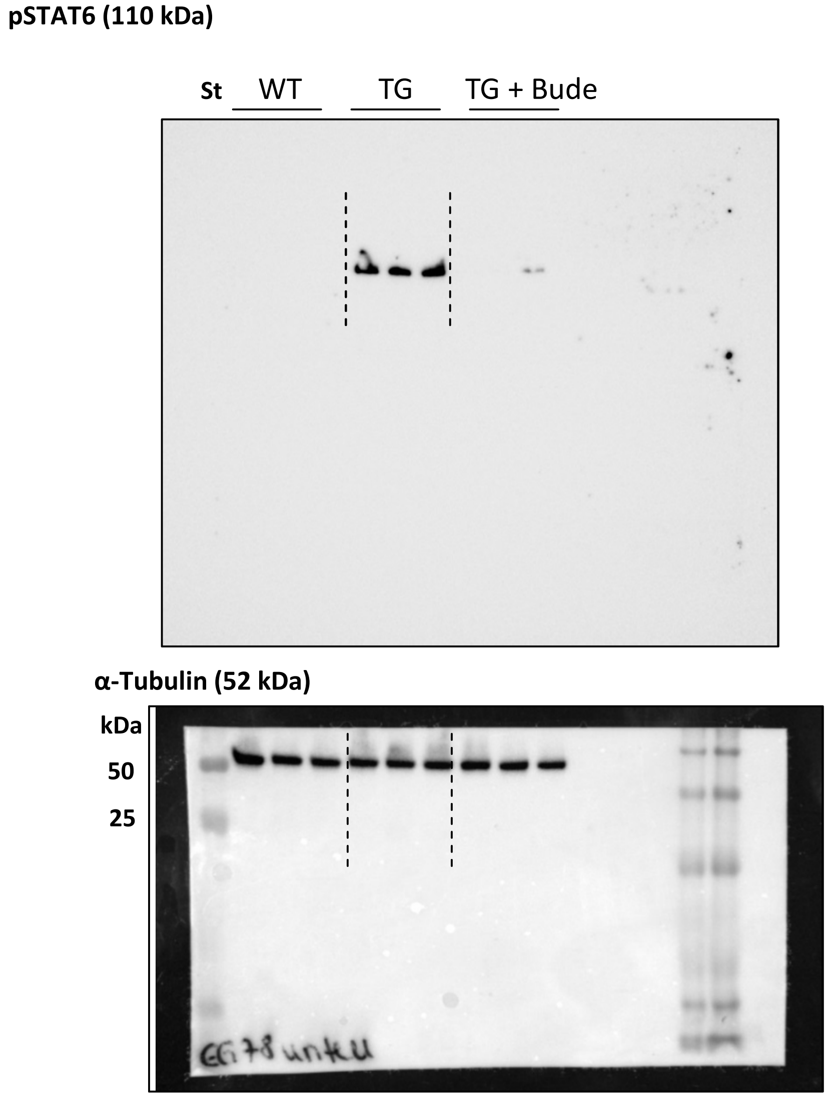

(Figure 9E)

Figure E5

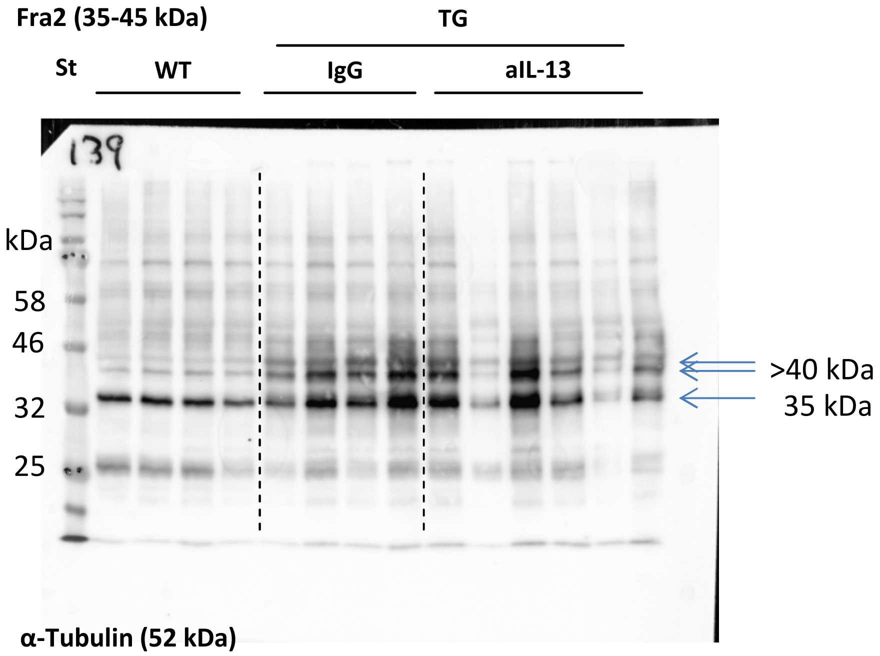

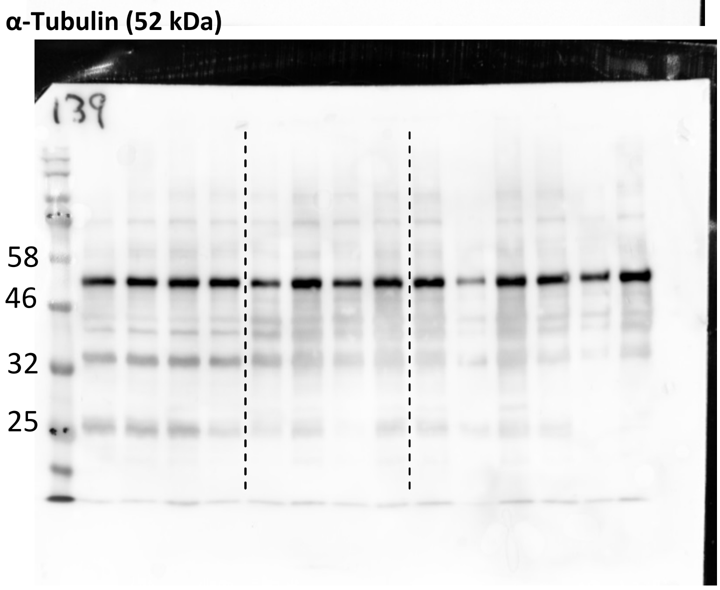

(Figure 10A)

Figure E6

Fra2 (35-45 kDa)

St WT TG TG + Bude

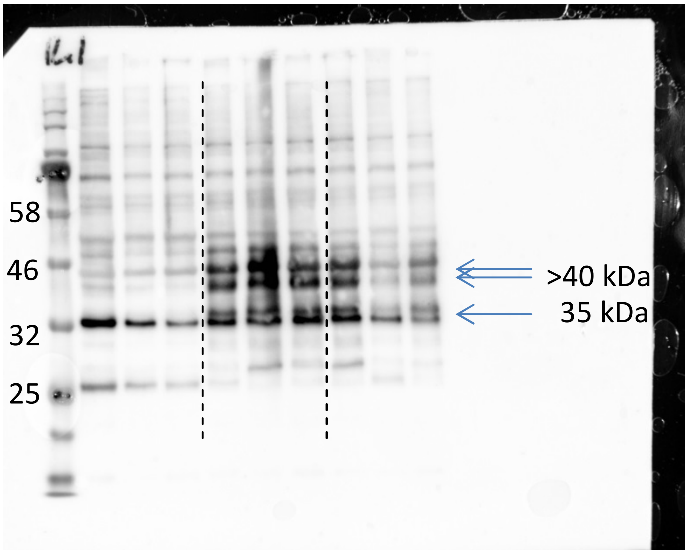

α-Tubulin (52 kDa)

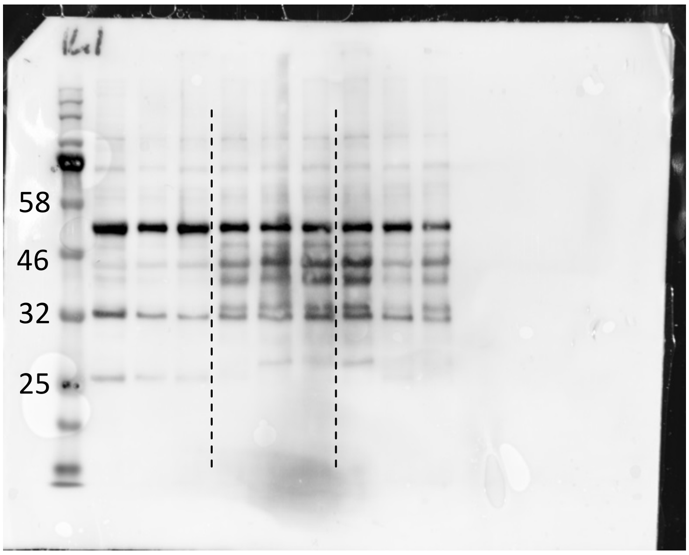

(Figure 10B)
